# Some National Aspects of Transport

**Published:** 1920-07-24

**Authors:** 


### SCIENCE AND THE NATION.

### Some National Aspects of Transport.

This was the subject of Lord Montagu's address at the recent British Science Guild meeting, and he explained many political and social problems which are closely bound up with transport. Nearly all our great transport agencies are unsatisfying. For some time past, all over the world, there has been scarcely a branch of transport, by land, sea, or air, which is paying a commercial return on the capital invested. The London General Omnibus Co. is now working at a loss. But in country, as opposed to urban, districts the outlook is not so gloomy as some transport companies, especially those conveying heavy goods, are paying their way. Even in shipping, authorities speak of a difficult time ahead. The wonderfully regular air service between England and the continent is being carried on at a loss. Therefore if the transport services are to continue, either the users of them will have to pay more, or the State —*i.e.*, the taxpayer or the ratepayer—will have to continue subsidies to transport undertakings. It is not surprising that we are building no new railways, and that the making of new roads is being readily undertaken, for congestion, on road and rail alike, is becoming more intense.

The State cannot, however, be looked to for permanent help; it is already too heavily burdened, and at the moment it cannot borrow at less than 6 per cent. It has been said that by the abolition of competition, such as several railway systems combining, and the employment of more scientific methods of handling traffic, it would be possible to save expense. There is the danger, however, that the saving might be largely counterbalanced by the expense of a department to carry out traffic control. There is now an increased tendency for traffic to leave the railway for the sea, the canal, and the road. The comparative cheapness of the road traffic for short distances has deprived the railways of much revenue already; and the time has arrived, therefore, when the question of having special roads for mechanical road traffic, from which all other traffic should be excluded, must be faced. The cheapness and superiority of rail transport is due to having a steel wheel on a steel rail, under which conditions 15 lb. traction will move a ton, whereas on a good tar-macadam road the tractive force required is three times as much, and it is increased to 100 lb. per ton on a muddy road. A plateway of steel and concrete could, said Lord Montagu, be laid down between London and Birmingham, the traction per ton on which would probably not exceed 20 lb. Very few buildings *en route* would be required, and the working expenses would be very small compared with those of the ordinary railway. Goods could then be conveyed from door to door without the present very expensive intermediate handling. Overhead roadways might be constructed between big towns and their suburbs. Roads specially built and reserved for mechanical vehicles are still believed to be the best immediate solution of the increasing suburban traffic difficulties. The speaker discussed the question of the great increase in local property values in the neighbourhood of a new railway, and thought a local transport benefit tax might be levied on those who became richer by the advent of such developments. When the "Tube" railway to Golders Green became operative, local property was enhanced in value some 300 per cent. to 400 per cent. !

It would certainly be a great gain to the country if some rich man would endow at Oxford or Cambridge a Chair of Transport, so that the science of transport could be *studied* apart from the disturbing influences of politics and trade disputes. The possibility of gas-suction plant being used to propel locomotives, motor lorries, and ships should be remembered; and the suitability of benzol and alcohol for the internal-combustion engine. As yet it is difficult to see much alteration in the Government's attitude towards science; and for many years Government Departments have been contemptuous on this matter. Nowadays we understand it is the intention of the British Science Guild to try to place its services at the disposal of the nation and the governing authorities. We wish them every success in an excellent cause.

Later the Rt. Hon. Lord Bledisloe, K.B.E., proposed the adoption of the annual report in a detailed speech, and pointed out, in urging the great cause of science, that Oxford had now an unprecedented number of students who were devoting themselves to science, and a greater number preparing to take their Final Honours in Science than in any other school of the University. As Oxford had not been in the past very closely identified with scientific enterprise, that was a fact of which there was every reason to feel proud. The report spoke of the need of scientific research as part of the equipment of our manufacturers if our successful competition with other countries was to be maintained. In the past, many of our promising young scientists had not found scope for their energies in this respect, and in consequence some had been lost to their country. The most interesting subject dealt with in the report was the proposed conference between this Guild and other representatives of science on the one hand, and those of the official Labour Party on the other, which it was hoped would take place in the next few months, so that some agreed standpoint could be arrived at in relation to scientific progress. It was hoped by the Council of the Guild that labour men could be convinced that the application of science to industry was all to their ultimate advantage. The Guild's appeal to the Foreign Office to appoint scientific *attachés* to the Embassies had not yet been attended to, but it was hoped that eventually it would be. It was hoped, by setting up a Committee to scrutinise Bills which had a scientific and technical side, to safeguard that aspect, and exercise an influence on the ultimate form of such Bills.

